# Spider phobia is associated with decreased left amygdala volume: a cross-sectional study

**DOI:** 10.1186/1471-244X-13-70

**Published:** 2013-02-26

**Authors:** Melanie S Fisler, Andrea Federspiel, Helge Horn, Thomas Dierks, Wolfgang Schmitt, Roland Wiest, Dominique J-F de Quervain, Leila M Soravia

**Affiliations:** 1 Department of Psychiatric Neurophysiology, University Hospital of Psychiatry, University of Bern, Bolligenstrasse 111 3000, Bern, 60, Switzerland; 2 Diagnostic and Interventional Neuroradiology, Inselspital and University of Bern, OP-Ost C215, Bern, 3010, Switzerland; 3 Division of Cognitive Neuroscience, Faculty of Medicine & Faculty of Psychology, University of Basel, Birmannsgasse 8 4055, Basel, Switzerland

**Keywords:** Spider phobia, Amygdala, Morphology

## Abstract

**Background:**

Evidence from animal and human studies imply the amygdala as the most critical structure involved in processing of fear-relevant stimuli. In phobias, the amygdala seems to play a crucial role in the pathogenesis and maintenance of the disorder. However, the neuropathology of specific phobias remains poorly understood. In the present study, we investigated whether patients with spider phobia show altered amygdala volumes as compared to healthy control subjects.

**Methods:**

Twenty female patients with spider phobia and twenty age-matched healthy female controls underwent magnetic resonance imaging to investigate amygdala volumes. The amygdalae were segmented using an automatic, model-based segmentation tool (FSL FIRST). Differences in amygdala volume were investigated by multivariate analysis of covariance with group as between-subject factor and left and right amygdala as dependent factors. The relation between amygdala volume and clinical features such as symptom severity, disgust sensitivity, trait anxiety and duration of illness was investigated by Spearman correlation analysis.

**Results:**

Spider phobic patients showed significantly smaller left amygdala volume than healthy controls. No significant difference in right amygdala volume was detected. Furthermore, the diminished amygdala size in patients was related to higher symptom severity, but not to higher disgust sensitivity or trait anxiety and was independent of age.

**Conclusions:**

In summary, the results reveal a relation between higher symptom severity and smaller left amygdala volume in patients with spider phobia. This relation was independent of other potential confounders such as the disgust sensitivity or trait anxiety. The findings suggest that greater spider phobic fear is associated with smaller left amygdala. However, the smaller left amygdala volume may either stand for a higher vulnerability to develop a phobic disorder or emerge as a consequence of the disorder.

## Background

Specific phobias are characterized by automatic, exaggerated fear responses towards phobia-specific objects [1]. Converging evidence implies the amygdala as the most critical structure involved in processing of phobia-relevant, but also general threatening stimuli [2-6]. Through its broad connections to other brain areas, it might be involved in mediating automatic responses to potential danger [7]. Due to its projections to the visual stream, it is further likely to modulate sensory processing [8]. In contrast to large functional imaging evidence for the involvement of the amygdala in fear, there have been few reported studies on its structural abnormality in mood and anxiety disorders [9-12]. Reduced amygdala volume (AMV) has been reported to be significantly correlated with the severity of distortion in anxiety [13,14] and panic disorder [15]. Structural brain imaging studies in specific phobia are lacking. This raises the question whether functional differences in phobic patients appear in association with structural differences. Because of evidence of hyperactivity and structural differences in anxiety disorders, we hypothesized that also spider phobic patients (SP) express reduced AMV compared to healthy controls. Phobic fear may be characterized by a combination of physiological and behavioral components. In order to clarify the contribution of possible confounding factors, several aspects of phobic disorders that could be critical to the pathogenesis have to be considered. Anxiety-related personality traits have been suggested to represent important predisposing factors for anxiety-related disorders [16,17]. Other potential moderating variables may be the severity and duration of the disease and disgust sensitivity. Therefore, the association between AMV and several clinical features has been investigated in this study. To the best of our knowledge, this is the first study investigating volumetric differences of the amygdala in SP compared to healthy control subjects.

## Methods

### Subjects

Twenty female patients with a current diagnosis of spider phobia and twenty healthy female controls matched for age were included in the analysis. Subjects were recruited via advertisements. The data used for this report has been collected in a larger project investigating the effect of cortisol on the outcome of an exposure-based short-term group therapy for spider phobia. Because the study design included an exposure task and in order to minimize motion artifacts, only patients that could keep still while facing a picture of a spider were included in the magnetic resonance paradigm. Exclusion criteria for patients were the following: any axis I other than specific phobia for spiders, axis II disorders, the manifestation of acute or chronic medical condition, neurological diseases, current drug or alcohol abuse or any contraindication to magnetic resonance imaging (metallic objects, pregnancy) or confounding factors for structural brain studies (regular medication, contraceptives, left handedness). Healthy control subjects were excluded from the study if they met any of the following exclusion criteria: axis I-disorders (measured with the SCL-90-R), the manifestation of acute or chronic medical condition, neurological diseases, current drug or alcohol abuse or any contraindication to magnetic resonance imaging (metallic objects, pregnancy) or confounding factors for structural brain studies (regular medication, contraceptives, left handedness). Informed written consent was obtained from all participants after description of the study, which was approved by the ethics committee of the Canton of Bern, Switzerland (161/07) in accordance with the principles of the Declaration of Helsinki [18]. Written informed consent was obtained from the patient for publication of this report and any accompanying images. A copy of the written consent is available for review by the Editor-in-Chief of this journal.

### Diagnostic instruments and questionnaires

Patients: Diagnoses for specific phobia for spider was based on the Diagnostic and Statistical Manual of Mental Disorders, fourth edition (DSM-IV) [1]. Specifically, we used a computer-based structured clinical interview (DIA-X) [19] which is based on the Composite International Diagnostic Interview (CIDI) [20]. Patients were screened for possible axis II disorders, respectively personality accentuation using SKID-II-questionnaire [21]. The SKID-II is an efficient, user-friendly instrument that helps to make standardized, reliable, and accurate diagnoses of the 10 DSM-IV Axis II personality disorders.

Control subjects: SCL-90-R was used as short screening to exclude Axis-I disorders (such as anxiety disorders and depression) in the control subjects [22].

All subjects filled out the German version of the Spider Phobia Questionnaire (SPQ) [23] and a questionnaire for the assessment of disgust sensitivity (FEE) [24]. The SPQ is a validated self-report questionnaire widely used for assessing spider phobic symptom severity. It consists of 31 items which could be answered by “true” or “false” statements. Subjects with a SPQ (range 0-31) score of less than 21 “true” statements were treated as healthy controls. The FEE is a further development of the English Disgust Scale [25]. It is used to measure individual differences in sensitivity to disgust, and to examine the relationships among different kinds of disgust. Humans with a high disgust sensitivity show longer and more intensive disgust reactions than those with lower disgust sensitivity. Disgust sensitivity is treated as a vulnerability factor for the development and maintenance of disorders such as phobias. Participants had to rate how disgusted they feel when confronted with several stimuli on a five-points Likert-scale (0 = ‘not disgusting at all’ to4 = ‘very disgusting’). A total score (range: 0–148) can be calculated which captures a measure for overall disgust sensitivity towards disgust-eliciting stimuli. State (both groups) and trait anxiety (only in patient group) were measured using the German version of the State-Trait Anxiety Inventory (STAI) [26]. The STAI consists of two 20-item scales for measuring the anxiety intensity as an emotional state and individual differences in anxiety proneness as a personality trait. The STAI state reports the intensity of anxiety feelings at the moment of assessment. Responses to the STAI trait items requests subjects to indicate how they generally feel. Both scales range from 20 to 80 points. Handedness was assessed via the Edinburgh Handedness Inventory (EHI) [27].

### Data acquisition

Magnetic resonance imaging was performed on a 3 T Siemens Magnetom Trio Scanner (Erlangen, Germany) equipped with a standard radio-frequency head coil. For structural images, a high-resolution 3D T1-weighted imaging protocol (modified driven equilibrium Fourier transform, MDEFT [28]) was used, resulting in 176 sagittal slices of 1.0 mm thickness, 256×256 mm field of view (FOV), and a matrix size of 256×256, resulting in a voxel size of 1x1x1mm. Further scan parameters were 7.92 ms repetition time (TR) 2.48 ms echo time (TE) and 910 ms inversion time (Ti) for an optimal contrast-to-noise ratio (see [28]). Subjects were measured within their luteal phase of their menstrual cycle, because a study in healthy female subjects revealed an increase in AMV during the premenstrual phase compared to the late follicular phase [29].

### Segmentation of the amygdala

**Figure 1:**
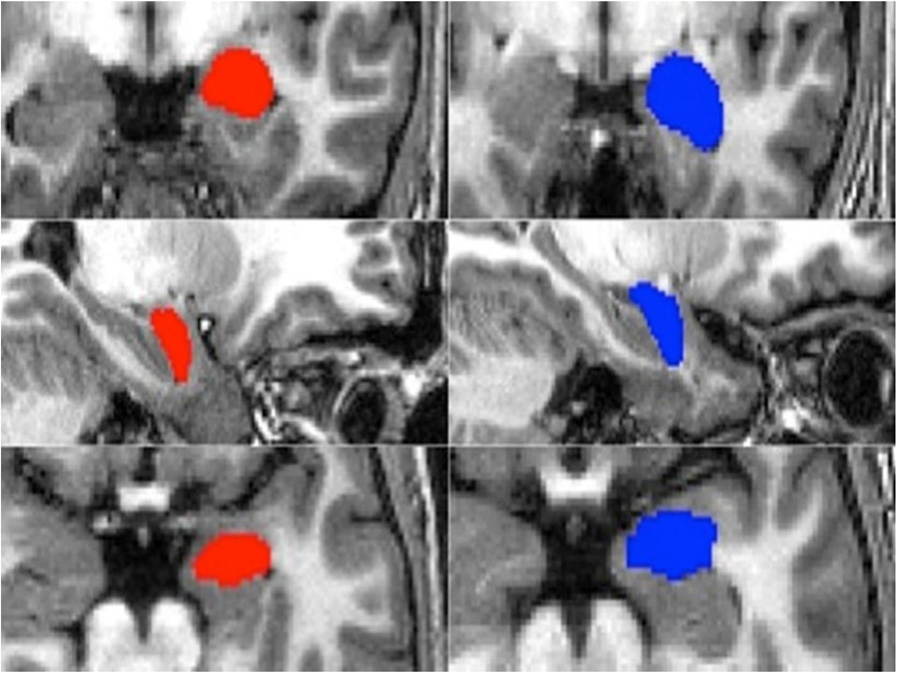
**Segmentation sample.** Example of the automatic amygdala segmentation with FSL_FIRST of one patient (red) and one control subject (blue): coronal, saggital, and axial views of the automatically set boundaries.

For subcortical segmentation, a fully-automated segmentation method (FSL FIRST; http://fsl.fmrib.ox.ac.uk/fsl/fslwiki/) was used [30], which has been shown to accurately segment subcortical structures [31]. The reliability for the segmentation of the amygdala by FIRST has been demonstrated and was correlated with those of manual tracing [32]. It is based on multivariate Gaussian shape/appearance models, which are constructed from a large set of manually labeled and segmented images (336 brains) from the Center for Morphometric Analysis (MGH, Boston) and uses a Bayesian framework that includes intra- and inter-structural variability. Using T1 images, the segmentation was performed with two stage affine transformation to standard space of MNI152 at 1 mm resolution. The first stage was a standard 12 degrees of freedom registration to the MNI152 template after which the normalization has been checked manually. The second stage applied 12 degrees of freedom registration using an MNI152 amygdala mask to exclude voxels outside this subcortical region. The segmented images were then used to produce mesh and volumetric outputs with boundary correction. Voxels exhibiting ambiguous structural characteristics, which are usually located at the borders between adjacent structures (partial volume effect), were classified as boundary voxels. The algorithm for the boundary correction requires the number of modes of variation (iterations) as input, which was set to 80 for the amygdalae (as recommended by Patenaude and colleagues [33]). The vertex information was then automatically transformed back to native space using the inverse transformation matrix where the boundaries were corrected. Finally, the summary images of the segmentation outputs were checked for quality and registration. Samples for the automatic segmentation of FIRST are shown in Figure 1.

### Brain volume measurement

To correct for variations in individual’s head size, the measured AMV were divided by the total intracranial volume (ICV). For each subject, the ICV was measured applying the software tool “BET” (Brain extraction tool) [34], provided as part of the FSL software distribution. The extracted voxels were then summed up to provide an estimate of the ICV.

### Statistical analysis

SPSS 19 software (SPSS Inc., Armonk, NY) was employed for statistical analyses. The following tests were used for the statistical analyses: two-sample independent t-tests to compare differences between groups for demographic, clinical and for brain volume variables; relative amygdala volume values (corrected for ICV) were used for the multifactorial ANCOVA with group as between-subject factor and left and right amygdala as the dependent factors; for the association between AMV and clinical features, we computed Spearman correlations and Wilcoxon’s test was applied for comparisons of variables which were not normally distributed (SPQ and STAI state pre). In order to check whether the data is normally distributed, the Mauchly-Test of Sphericity was applied. The effects of BMI and age on AMV were examined with separate regression analyses with BMI or age as independent variables and left or right amygdala volume as dependent variables. For all statistical analyses, p values of <.05 (two-sided) were considered as significant results.

## Results

### Demographic and psychological measures

Patients and control subjects did not differ significantly with respect to any demographic variables (Table 1). The age of the subjects ranged from 20 to 54 years with a mean of 29.9 years in patient group (*SD* ± 11.3) and 27.1 years in control group (*SD*± 5.9). Group analysis revealed that patients showed significant higher mean scores on the SPQ (W=214.5, Z= -5.30, p=0.00). Furthermore, patients reported higher state anxiety (STAI-state) before scanning (which included also exposure to spiders; *W= 211.00, Z= -1.97, p= 0.05*), but not after scanning (*F [*3,36*]*= 6.32; *p*= 0.25), suggesting patients suffering from anticipatory anxiety.

**Table 1:** Demographic and clinical characteristics of the patients and their control group

	**Patients (n=20)**		**Controls (n=20)**					
**Characteristics**	**Mean**	**SD**	**Mean**	**SD**	**df**	**t**	**p**	**Equality of variance**
Age (years)	29.9	11.3	27.1	5.9	38	−0.98	0.33	0.91
Age at onset	6.65	3.28	-------	-------	-------	-------	-------	--------
Duration of illness (years)	22.14	13.01	-------	-------	-------	-------	-------	-------
Handedness scores	9.7	0.58	9.8	0.41	38	0.65	0.52	0.98
SPQ	21.55	4.29	6.1	3.83	38	−11.99	0.00	0.00
FEE	85.3	22.93	73	26.42	38	−1.47	0.15	0.17
STAI state pre / post scanning	39.85/ 34.85	10.67/ 12.9	32.5/ 30.53	4.05/ 6.61	38	−2.51/ -1.18	0.05/ 0.24	0.05/ 0.54
STAI trait	44.8	6.89	-------	-------	-------	-------	-------	-------
BMI	21.8	3.90	21.63	2.31	38	−1.18	0.87	0.61

SPQ: Spider Phobia Questionnaire; FEE: disgust sensitivity scale; BMI: body mass index.

#### Amygdala volume

**Figure 2:**
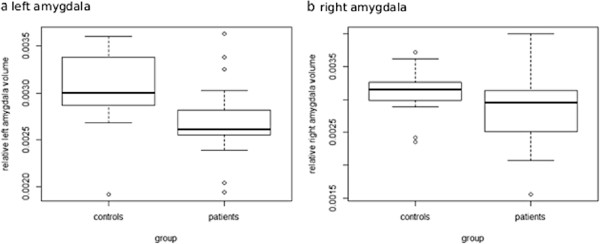
**Amygdala volumes of spider phobic patients and healthy controls.** The central box shows the data between 25^th^ and 75^th^ quartiles, with the median represented by the line. The whiskers extend from the upper and lower quartiles to a distance of 1.5 interquartile range (IQR). Circles represent the outliers over 3 IQR below the 25^th^ or above the 75^th^ quartile. Relative amygdala volumes were calculated by the following formula: 100x(absolute amygdala volume in mm^2^/intracranial volume in mm^2^). **a**: boxplot comparing relative left amygdala volume; **b**: boxplot comparing relative right amygdala volume.

**Figure 3:**
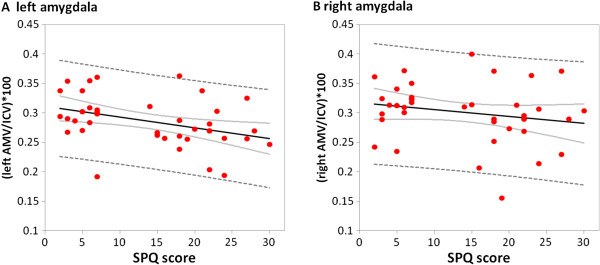
**Relation between the amygdalae and Spider Phobia Questionnaire (SPQ) scores. A**: The scores on the SPQ are negatively correlated with the left amygdala volume (r=-0.425, p=0.011). More phobic symptoms are found in individuals who have smaller left amygdala. **B**: The volumes of the right amygdala are not significantly correlated with SPQ Scores.

Multifactorial ANCOVA of AMV, adjusted for ICV, showed approximately 13% smaller AMV in patients on the left than in controls, resulting in a significant between group effect for left AMV (*F [*3,36*]*= 6.39; *p*= 0.02; Figure 2a). Separate regression analyses indicated that the difference in left AMV between patients and controls were not accounted for by differences in age (*F*= 1.72; *df*= 36, *p*=0.20) and BMI (*F*= 0.55, *df*= 36, *p*= 0.47). There was no significant difference of right AMV between patients and controls (*F [3,36]* = 2.28; *p*= 0.20; Figure 2b). Within the whole group, SPQ scores were negatively correlated with left AMV (*r*=-0.47; *p*=0.005; Figure 3). Separate regression analyses showed that smaller left amygdala occurred independently of age (*r*=-0.046; *p*=0.79) and BMI (*r*=-0.143; *p*=0.42). Correlations between left amygdala and the disgust score (FEE) or state anxiety (STAI trait/state) did not reach significance, nor were there any significant correlations between right AMV and clinical scores within the whole sample. Within the phobic sample, trait anxiety did not correlate with AMV. Thus, differences in AMV did not appear to be due to age or BMI or clinical features of the participants.

## Discussion

The purpose of this study was to determine whether there is evidence for AMV differences in patients with spider phobia compared to healthy controls. As hypothesized, the findings confirm a reduction in left AMV of patients. Furthermore, the reduced left AVM is associated with the severity of spider phobic symptoms. Our finding of abnormal left AMV in patients are consistent with findings that report diminished amygdala size in fear and anxiety-related disorders [10,35-40]. We suggest that the automatic manifestation of fear responses is mediated by a potentiated fear network which may be associated with an amygdala deficiency.

However, the differences of AMV could be also influenced by clinical factors. The present findings show an association between left AMV and symptom severity in phobic patients in the way that the smaller the left amygdala, the more phobic symptoms they reported. This finding seems to be attributed to spider phobia, because we selected patients with pure spider phobia without any comorbidities and no other potential confounding factor has shown to be associated with this volume reduction in spider phobic patients. It is worth remarking that findings of smaller left AMV, as reported in this study, suggest that abnormal functioning of this structure may underlie the symptoms of automatic and exaggerated fear response observed in specific phobias, as measured with the SPQ, which reliably assesses fear of spiders [41,42].

Hemispheric differences in amygdala alterations found in this study may potentially be in line with different roles in emotion processing for the left and right amygdala which has been found in several studies investigating mood and anxiety disorders [39,43]. Based on these studies, it is assumed that the left amygdala is more involved in processing sustained stimulus evaluation while the right one is more involved in rapid and undifferentiated processing of emotional stimuli [44].

Some limitations of the present study need to be mentioned. We only studied female subjects; therefore the results cannot be generalized to men. We should further mention the different diagnostic measurements for the control and patient group. The patient group was additionally screened for possible personality disorders (SKID-II) and trait-anxiety (STAI-trait). Whereas the control subjects were only screened for possible axis I-disorders (SCL-90-R) which implied exclusion of the study. Additionally, the sample size was modest and should be extended. However, the patient group can be considered to be homogenous, as patients did not suffer from any other axis I disorders at the time of assessment. Hence, future investigations should include comparisons of amygdala morphology in various types and degrees of phobic disorders for a better pathophysiological distinction. Longitudinal and pre-post treatment studies should clarify the meaning of the observed amygdala differences over time.

## Conclusion

Still, the reasons for volumetric differences are so far largely unclear. Two possible interpretations might be offered: First, smaller left AMV might be a vulnerability factor for the development of spider phobia. The second interpretation relates to experience or exposure-related structural modifications within the amygdala. Whether the observed atrophy of the amygdala in mood and anxiety disorders is progressive and already present at time of disease onset or develops as a result of damage secondary to higher amygdala activity is a matter of debate [45,46].

## Abbreviations

AMV: Amygdala volume; ANCOVA: Analysis of covariance; BMI: Body mass index; EHI: Edinburgh handedness inventory; FEE: Questionnaire for the assessment of disgust sensitivity; FOV: Field of view; ICV: Intracranial volume; SD: Standard deviation; SP: Spider phobic patients; SKID-II: Structured clinical interview for DSM-IV Axis II disorders; SPQ: Spider phobia questionnaire; STAI: State-trait anxiety inventory.

## Competing interests

The authors declare that they have no competing interests.

## Authors’ contribution

MF participated in the design of the study, carried out the measurements, performed the statistical analysis and drafted the manuscript. AF carried out the sequence alignment and revised the manuscript critically. HH participated in the diagnostic interview and revised the manuscript critically. TD revised the manuscript critically. WS participated in the diagnostic interviews. DQ made the conception and design of the study and revised the manuscript critically. LS participated in the design of the study and revised the manuscript. All authors read and approved the final manuscript.

## Pre-publication history

The pre-publication history for this paper can be accessed here:

http://www.biomedcentral.com/1471-244X/13/70/prepub
